# The effect of moderate exercise on the elevation of Bax/Bcl-2 ratio in oral squamous epithelial cells induced by benzopyrene

**DOI:** 10.14202/vetworld.2018.177-180

**Published:** 2018-02-13

**Authors:** Anis Irmawati, Nadira Jasmin, Sidarningsih

**Affiliations:** 1 Department of Oral Biology, Faculty of Dental Medicine, Universitas Airlangga, Surabaya, Indonesia; 2 Undergraduate student of Faculty of Dental Medicine, Universitas Airlangga, Surabaya, Indonesia

**Keywords:** Bax/Bcl-2 ratio, benzopyrene, moderate exercise, oral squamous carcinoma

## Abstract

**Aim:**

The aim of this study was to analyze the effect of moderate exercise on the elevation of Bax/Bcl-2 ratio.

**Materials and Methods:**

Eighteen *Mus musculus* strain Swiss Webster (Balb/c) were divided into three groups (n=6). K1 and K2 had contact with water 3 times/week for 12 weeks, while the members of the K3 group swam 3 times/week for 12 weeks while carrying load weighed 3% of their body weight. After 5 weeks, they were induced with 0.04 ml oleum olivarum (K1), 0.08 mg benzopyrene/0.04 ml oleum olivarum (K2, K3) 3 times/week for 4 weeks. Immunohistochemistry assays were used to determine the ratio of Bax/Bcl-2 expression. The results were analyzed using an independent t-test.

**Result:**

The Bax/Bcl-2 ratio increased significantly in K3 compared to K2 (p=0.00).

**Conclusion:**

Moderate exercise could increase the Bax/Bcl-2 ratio in oral squamous epithelial cells induced by benzopyrene.

## Introduction

Cancer is a leading cause of death worldwide. Data from the Global Cancer Control conducted by the International Agency for Research on Cancer suggests that in 2012 there were 14,067,894 new cases of cancer and 8,201,575 cases of cancer-related death. According to *Riset Kesehatan Dasar* (riskesdas), in 2013 the prevalence of cancer in the population of all ages in Indonesia amounted to 1.4%. Meanwhile, the percentage of oral cavity and lip cancer cases worldwide in 2012 represented 4.0% of new cases with 1.9% mortality rate [1]. Around 90% of oral cancer malignancy consists of squamous cell carcinoma [2]. The incident rate of smoking, the biggest risk factor for oral cavity cancer, is still high in Indonesia. According to Tobacco Atlas 2007, Indonesia was the fourth-ranked country in terms of tobacco consumption [3]. One cigarette compound, polycyclic aromatic hydrocarbons (PAHs), is carcinogenic. Benzopyrene is one PAH compound that has been widely studied. It is metabolized by oxidative enzymes, especially cytochrome p450 [4]. After going through several stages of metabolism, benzopyrene will produce carcinogenic metabolites, e.g. Benzopyrene 7,8 diol-9,10-epoxide (BPDE) which cause mutant cells formation [5].

Apoptosis is the ability to eliminate mutated cells which can lead to cancer. There are three Caspases activation pathways, one of which is the intrinsic pathway (mitochondrial). This is controlled by a group of proteins belonging to the Bcl-2. Bcl-2 protein group consists of anti-apoptotic proteins such as Bcl-2, Bcl-XL, and the pro-apoptotic protein, e.g. Bax [6]. Bax is an initiator protein of apoptosis through the formation of Mitochondrial Membrane Permeabilization [7]. In contrast, Bcl-2 has the opposite function of inhibiting apoptosis and maintaining cells [8,9]. Activation of Bax expression will inhibit that of Bcl-2 [9,10]. The ratio of the two protein expressions can affect the ongoing absence of apoptosis as the increases in the Bax/Bcl-2 ratio considered to be a reliable indicator of cells to undergo apoptosis [10,11].

At present, the treatment of cancer still centers on herbal or chemical remedies, the study of cancer prevention remaining very rare. Physical exercise has many benefits such as reducing the risk of systemic diseases, i.e. heart disease, hypertension, diabetes, and osteoporosis [12]. Physical exercise can also affect immunity depending on its frequency, duration, and intensity [13]. Therefore, this study aims to analyze the effects of physical exercise as a method of preventing cancer by investigating the influence of moderate-intensity physical exercise in the elevation of Bax/Bcl-2 ratio in oral squamous epithelial cells induced by benzopyrene.

## Materials and Methods

### Ethical approval

Ethical approval for the study was obtained from the Board for Animal Experiments at the Faculty of Dental Medicine, Universitas Airlangga, No. 156/KKEPK.FKG/VIII/2016.

### Animals

The sample used consisted of 18 male mice of the *Mus musculus* Swiss Webster strain (Balb/c), aged±2 months, weighing 20-40 g and equally divided into three groups: A negative control group (K1), a positive control group (K2), and a treatment group (K3).

### Experimental design

This research represented a laboratory post-test only control group design. The *M. musculus* was given 7 days to acclimatize. K1 and K2 experienced contact with water for 70% of maximal work capacity time, 3 times/week for 12 weeks, while K3 was allowed to swim 3 times/week for 12 weeks, while carrying a load weighed 3% of their body weight. After 5 weeks, K1 was induced with 0.04 ml oleum olivarum on the top right buccal mucosa of the oral cavity 3 times/week for 4 weeks. During the same period, K2 and K3 were induced with 0.08 mg benzopyrene (Sigma-Aldrich, Saint Louis, Missouri, USA)/0.04 ml oleum olivarum on the top right buccal mucosa of the oral cavity 3 times/week for 4 weeks.

### Immunohistochemistry assay

During week 13, all animal subjects were anesthetized with ether before being sacrificed to obtain a sample of upper right buccal mucosa tissue. The samples were then fixed in 10% buffered formalin and made into a paraffin block. Immunohistochemistry staining was performed using monoclonal Bax antibodies from Dako (Agilent, Santa Clara, and USA) and Bcl-2 (Santa Cruz, California, and USA). The expression of Bax and Bcl-2 were subsequently calculated under a light microscope (Olympus, Tokyo, and Japan) at 10 different visual fields at 400× magnification.

### Statistical analysis

The data obtained were analyzed by means of SPSS version 20 (IBM, New York, and USA) using an independent t-test to determine the differences between the two groups (K2 and K3) because the expression of Bcl-2 in K1could not be found (Bcl-2=0).

## Results

**Figure-1:**
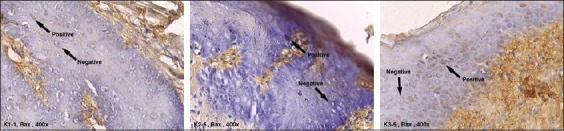
The expression of Bax in K1, K2, and K3 (arrows indicate positive cells expressing Bax: Brown color).

**Figure-2:**
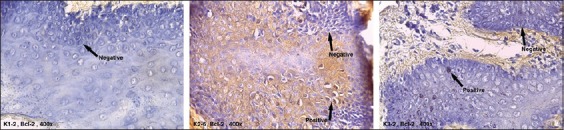
The expression of Bcl-2 in K1, K2, and K3 (arrows indicate positive cells expressing Bcl-2: Brown color).

The expression of Bax was found in all groups (Figure-1). In contrast, the expression of Bcl-2 was only found in K2 and K3 (Figure-2). The contents of Table-1 show that the mean of Bax/bcl-2 ratio in K1 could not be defined due to the negative expression of Bcl-2 in that group, while there was a significant increase in Bax/bcl-2 ratio expression in K3 compared to K2 (p=0.000).

**Table-1:** Mean and standard deviation of Bax/Bcl-2 ratio expression.

Group	n	Bax/Bcl-2 ratio	Independent t-test (p)

		Mean±SD	
K1	6	-	0.000*
K2	6	0.32±0.23	
K3	6	1.10±0.17	

## Discussion

Induction of benzopyrene can form carcinogenic metabolites called BPDE which develop the covalent bonds with p53 DNA, resulting in a mutation in p53 DNA. When mutations occur in some loci of p53, the unmutated p53 loci will be phosphorylated. The phosphorylation of p53 loci causes the detachment of active p53 and MDM2 bonds [14]. The active p53 can trigger the transcription of DNA Bax, producing an increased level of Bax protein synthesis [15]. p53 also regulates apoptosis-associated speck-like protein (ASC) and subsequently assists in the activation of Bax and Bax interaction with mitochondria [7].

The increased level of Bax will lead to the translocation of Bax from cytosol to the outer membrane of mitochondria, the formation of supramolecular openings either alone or together with other apoptotic proteins such as Bak or tBid, and the increased permeability of the inner mitochondrial membrane [7,10]. The increased permeability causes swelling of the matrix which eventually leads to outer mitochondria membrane breakdown and spillage of cytochrome-c. In the cytosol, cytochrome-c interacts with adaptive molecules, namely, Apaf-1, which leads to the activation of pro-caspase 9 into caspase 9 and activation of pro-caspase 3 and 7 to induce apoptosis [11,16]. p53 can also induce Bax to commence apoptosis which suppresses irregular cell growth such as cancer [9,10]. It is implied that if the mutant cells undergo apoptosis, such transformed cells are not expected to be formed.

Moderate exercise can increase the Bax/Bcl-2 ratio, confirmed by the high level of Bax/Bcl-2 ratio found in K3. The results of this study were contrary to those of Haack *et al*.[17] which stated that voluntary physical activity and chronic stress inhibits the increased expression of Bax. On the other hand, this finding is in line with research conducted by Marfe *et al*.[18] suggesting that there is an increased expression of Bax in amateur runners who participated in a 60-min marathon run.

Moderate exercise will open Ca^2+^ channels in the cell membrane and cause Ca^2+^ influx which increases the concentration of Ca^2+^ within the cell. The increasing level of Ca^2+^ will activate the mitogen-activated protein kinase (MAPK) through the bonding of Src signal transduction and RasGAP activation. The active MAPK will then translocate into the nucleus and activate the transcription of mRNA [19,20].

The results of this study also show that the largest expression of Bcl-2 was found in K2 because of the low expression of Bax in that group, as a result of the continuous induction of benzopyrene without any moderate exercise being performed. Moderate exercise could increase the expression of Bax that affects the suppression of Bcl-2 expression [21]. The role of Bcl-2 in the mechanisms of apoptosis is to retain cells and inhibit apoptosis [8]. At first, Bcl-2 is located in the cytoplasm and mostly associated with mitochondria, endoplasmic reticulum, and the cell nucleus envelope. The anti-apoptotic function of Bcl-2 plays a dominant role in the mitochondria. FKBP38 binds to and herds Bcl-2 from the endoplasmic reticulum and nucleus sheaths into the mitochondria. When Bcl-2 is translocated to the outer membrane of the mitochondria, Bcl-2 is capable of forming a heterodimer by binding with pro-apoptotic proteins such as Bax. The heterodimer formation may prevent apoptosis by blocking the mitochondria pores, thereby preventing the release of cytochrome C, caspase-9, and caspase-3 [22]. Activation of Bax expression will suppress Bcl-2 expression by binding to the domain BH1, BH2, and BH3 [23,24]. Because there is limited or no synthesis of Bax in K2, the synthesis of Bcl-2 is unrepressed. In K3, although the induction of benzopyrene was produced by means of the same dose and duration, the expression level of Bcl-2 was not high since the mice were performing moderate exercise resulting in a high level of Bax.

From the foregoing discussion, it can be concluded that the increase in the ratio of Bax/Bcl-2 matched the mechanism of apoptosis, as the process of apoptosis in the intrinsic pathway occurs because of the expression of Bax being higher than Bcl-2. This, in turn, results in the increasing ratio of Bax/Bcl-2 and initiates the release of cytochrome-C from the cytoplasm to form a complex apoptosome together with Apaf-1. This is then forwarded to induce caspase-9, along with caspase-3, the activation of which will cause apoptosis [22]. The increase in the Bax/Bcl-2 ratio also corresponds with the findings of previous research conducted by Jafari *et al*. which suggested that the form of physical exercise training undertaken by rats for 12 weeks (treatment with a daily 5-10 min run) can increase the ratio of Bax/Bcl-2 in cardiac muscle [25].

Finally, the influence of moderate exercise has been proven to increase the ratio of Bax/Bcl-2. Therefore, it is suggested that moderate exercise may prevent the onset of malignant processes. Although humans possess an independent apoptosis mechanism, it is anticipated that moderate exercise will help to increase the eradication of mutated cells.

## Conclusion

Moderate exercise could increase the Bax/Bcl-2 ratio in oral squamous epithelial cell induced by benzopyrene. We will make the further research in regard to this topic using other techniques.

## Authors’ Contributions

AI, NJ, and S collectively design the research. NJ carried out the literature, the experiment, and the data acquisition. AI carried out the statistical analysis. AI, NJ, and S collectively did the manuscript preparation, editing, and review. All authors read and approved the final manuscript.
